# Psychometric properties of the Chinese version of Sport Anxiety Scale-2

**DOI:** 10.3389/fpsyg.2023.1260253

**Published:** 2023-10-31

**Authors:** Jinrui Zhang, Zhiwen Zhang, Shuo Peng, Arsaythamby Veloo, Richard Peter Bailey, Wee Hoe Tan

**Affiliations:** ^1^Department of Physical Education, Neijiang Normal University, Neijiang, China; ^2^School of General Education, Chongqing Institute of Engineering, Chongqing, China; ^3^Faculty of Social Sciences and Liberal Arts, UCSI University, Kuala Lumpur, Malaysia; ^4^Faculty of Arts and Physical Education, The Open University of Henan, Zhengzhou, China; ^5^School of Education and Modern Languages, Universiti Utara Malaysia, Sintok, Bukit Kayu Hitam, Malaysia

**Keywords:** Sport Anxiety Scale-2 (SAS-2), cross-cultural adaptation, psychometrics, confirmatory factor analyses, competitive anxiety

## Abstract

**Introduction:**

The Sport Anxiety Scale-2 (SAS-2) is a validated measure of sports trait anxiety, with promising psychometric properties. However, its cross-cultural applicability in Chinese samples remains unexplored. Thus, the primary objectives of this study were twofold: to translate the SAS-2 into Chinese and assess the psychometric properties of the Chinese version.

**Methods:**

In Study 1, we initiated the translation of the SAS-2 into Chinese. This assessment involved bilingual Chinese students proficient in both English and Chinese. Additionally, we conducted a cross-linguistic measurement invariance analysis. In Study 2, we delved into the psychometric properties of the Chinese SAS-2 using a sample of Chinese student athletes. This examination encompassed an evaluation of its factor structure, convergent and discriminant validity, and measurement invariance across genders.

**Results:**

Our findings in Study 1 indicated no significant differences in item scores between the Chinese SAS-2 and the English version, and measurement invariance across languages. In Study 2, we uncovered that the Chinese SAS-2 and its factors exhibited excellent reliability, with Cronbach’s alpha values exceeding 0.80. Confirmatory factor analyses upheld the original three-factor model, demonstrating acceptable model fit indices (CFI = 0.96, TLI = 0.93, RMSEA = 0.08). Furthermore, all three factors of the Chinese SAS-2 displayed significant and positive correlations with athlete burnout and State-Trait anxiety. Additionally, this study elucidated the mediating role of Concentration Disruption (Somatic anxiety and Concentration Disruption) in the relationship between the Trait (State) anxiety, and athlete burnout. Moreover, we identified measurement invariance of the Chinese version of the SAS-2 across genders. Finally, female college athletes exhibited significantly higher scores in somatic anxiety and worry compared to their male counterparts.

**Discussion:**

In sum, our findings affirm that the Chinese version of the SAS-2 demonstrates robust reliability and correlates effectively with related criteria, thus validating its suitability for use in a Chinese context.

## Introduction

Anxiety is an emotion characterized by unpleasant feelings of tension, worrying thoughts, and corresponding physical symptoms (Spielberger, 2013). For example, in response to a perceived threat, the body typically mobilizes itself by tensing muscles, increasing breathing rate, and raising heart rate. Anxiety is not only closely related to well-being (Shek, 1993; Weidman et al., 2012) but also exerts a significant impact on our cognitive abilities (Eysenck and Calvo, 1992; Maloney et al., 2014) and sports activities (Krohne and Hindel, 1988). For instance, previous meta-analyses examining the relationship between anxiety and athletic performance have consistently found a significant and negative correlation. Furthermore, this meta-analysis has identified gender and age as moderators influencing the magnitude of this effect (Woodman and Hardy, 2003).

### Competitive anxiety

Within the realm of sports, athletic competitions tend to be inherently anxiety-inducing and feel stressed. For example, previous research conducted by Di Corrado et al. (2021) has found that psychological stress in elite canoe polo players plays a fully mediating role in mood and performance. As a result, anxiety has become a prominent area of focus within sports psychology, garnering attention from numerous researchers (Woodman and Hardy, 2003), particularly in relation to anxiety and sports performance. Competitive anxiety refers to the sport-specific anxiety experienced by individuals before or during competitive events (Martens, 1977). Researchers have also developed measurement instruments specifically tailored to assess trait anxiety in sports, aiming to quantify competitive anxiety (Martens, 1977; Smith et al., 1990).

Martens (1977) was the first to develop a unidimensional measurement tool called The Sport Competition Anxiety Test (SCAT). Subsequent studies have demonstrated the SCAT’s good reliability and validity across various samples (Cheatham and Rosentswieg, 1982; Brand et al., 1988). However, it is worth noting that the SCAT does not differentiate between somatic and cognitive anxiety or measure the differences between them. Afterward, Martens et al. (1980) developed the Competitive State Anxiety Inventory (CSAI) to evaluate self-confidence, as well as the physical and cognitive aspects of anxiety linked to a forthcoming competition. To comprehensively evaluate the multifaceted nature of anxiety, Martens et al. (1990) introduced the Competitive State Anxiety Inventory-2 (CSAI-2). This inventory was designed to gauge cognitive anxiety, somatic anxiety, and self-confidence. In addition, researchers developed the Sport Anxiety Scale (SAS) and its subsequent version, the SAS-2, as multidimensional measures of sport-specific trait anxiety. The SAS were based on the antecedents and consequences of cognitive and somatic anxiety (Smith et al., 1990, 2006). In the CSAI-2 and SAS-2, the CSAI-2 emphasizes the situational occurrence of the phenomenon, while the SAS-2 concentrates on sport-specific trait anxiety. Therefore, the present study will concentrate on the SAS-2, which assesses sport-specific trait anxiety, instead of the CSAI-2.

### The Sport Anxiety Scale (SAS)

Regarding the SAS, Smith et al. (1990) created a 21-item version of the scale, drawing from the cognitive-emotional model of anxiety and relevant empirical research. The development process involved exploratory and confirmatory factor analyses for cross-validation purposes. The scale was employed to assess an individual’s somatic anxiety, worry and concentration disruption. A previous study demonstrated a negative correlation between concentration disruption scores and the performance of college football players throughout a season (Smith et al., 1990). The multidimensional SAS may contribute to a clearer definition of sport-related anxiety and provide a more comprehensive understanding of the sport anxiety profile.

However, subsequent research revealed that the SAS might not be suitable for younger age groups (Smith et al., 1995). As a result, Smith et al. (2006) further revisions and developed the SAS-2, which comprises 15 items. Through exploratory and confirmatory factor analyses, the study successfully replicated the original SAS factor structure across various age levels. Notably, in the 9-to 10-year-old sample, three distinct subscales emerged: Somatic Anxiety, Worry, and Concentration Disruption. Importantly, the scale reliably predicted pre-competition state anxiety scores and demonstrated sensitivity to anxiety reduction interventions aimed at youth sport coaches (Smith et al., 2006). The SAS-2 has also exhibited strong psychometric properties in both child and adult samples, further validating its efficacy (Grossbard et al., 2007, 2009).

### Cross-cultural study of the SAS-2

As a result, the SAS-2 has been translated into different languages and its psychometric properties have been validated by researchers in several countries (Ramis et al., 2010; Silva-Rocha et al., 2019; Tomczak et al., 2022). As an example, Ramis et al. (2010) translated the SAS-2 into Spanish and determined that the Spanish version demonstrated good psychometric properties among child and adolescent athletes [Three-factor structure confirmatory factor analysis (CFA) model fit index: comparative fi t index (CFI) = 0.98, tucker-Lewis index (TLI) = 0.99, root-mean square standard error of approximation (RMSEA) = 0.05]. Following that, Silva-Rocha et al. (2019) examined the psychometric properties of the Brazilian version of the SAS-2 in professional and amateur athletes. The study’s findings revealed that the fit indices for the original three-factor model (Smith et al., 2006) did not reach a satisfactory level (CFI = 0.91, TLI = 0.90, RMSEA = 0.125). Subsequently, the correlation between the item 6 and item 12 errors was introduced into the original three-factor model, resulting in a notable improvement in the model’s fit indices (CFI = 0.97, TLI = 0.96, RMSEA = 0.08). Moreover, Ramis et al. (2015) collected data on three versions of the SAS-2 in Spain, Belgium, and Portugal, involving 842 athletes. The study found that the SAS-2 factorial model demonstrated invariance across gender, age, and sport type, underscoring its robustness and consistency across these variables. Moreover, CFI and TLI consistently exceeded 0.95, while RMSEA remained below 0.04 in the three-factor correlation CFA model.

Within the East Asian cultural context, the SAS-2 has been translated into different versions, such as the Malaysian version (Hashim et al., 2017), the Korean version (Cho et al., 2018), and the Indonesian version (Putra et al., 2021). These studies have found that the different language versions of the SAS-2 exhibit good psychometric properties within their respective languages. For instance, in the Hashim et al. (2017) study, the model fit indices for CFA were CFI = 0.93 and RMSEA = 0.06. Similarly, in the Putra et al. (2021) study, the CFA model exhibited CFI = 0.923, TLI = 0.91, and RMSEA = 0.08. As a result, they are considered valid and reliable measures for assessing anxiety levels. However, to date, there is a lack of cross-cultural validation of the SAS-2 among Chinese samples. Importantly, no study has translated the complete SAS-2 into Chinese and examined its applicability in China, which is the largest among the East Asian countries.

### The present study

As previously mentioned, there is a lack of validation for the SAS-2 among Chinese samples. It is still uncertain whether the Chinese version of the SAS-2 exhibits similar psychometric properties to the English version and whether it can effectively be applied to a Chinese sample. Therefore, the primary objective of the present study was to translate and cross-validate the psychometric properties of the SAS-2 in a Chinese sample. In Study 1, drawing upon prior cross-cultural research on scale instruments, we initially crafted and validated the Chinese version of the SAS-2 through a test–retest study involving a bilingual sample (Shou et al., 2017). Additionally, we conducted a multi-group CFA to assess measurement invariance between the Chinese and English SAS-2 versions (Ramis et al., 2015).

In Study 2, we scrutinized the psychometric properties of the Chinese version of the SAS-2 in a large sample of Chinese student-athletes. Firstly, we conducted an analysis to assess the reliability of the Chinese SAS-2 version. Subsequently, we examined the outcomes of fitting the original three-factor model using CFA. Furthermore, we delved into investigating the convergent and discriminant validity of the SAS-2 concerning State–Trait anxiety and athlete burnout. Lastly, we explored gender-based score differences and evaluated measurement invariance.

## Study 1

Study 1 aimed to translate the SAS-2 from English to Chinese, with the objective of ensuring the accurate reflection of the English version in the Chinese translation. To achieve this, the study examined the convergence between the English and Chinese versions by analyzing correlations and mean differences among the items. Furthermore, we conducted a multi-group CFA to assess measurement invariance across languages. A bilingual Chinese-English sample was utilized for this analysis.

### Participants and procedures

Study 1 consisted of 91 bilingual university students who voluntarily participated in the research. Among them, 77 were females. The participants had an average age of 19.63, with a standard deviation of 1.50. All these students were majoring in English. Prior to the start of the study, participants signed an informed consent form. In the classroom setting, they completed both the English and Chinese versions of the Sport Anxiety Scale-2. The order of administering the English and Chinese versions was balanced across participants. Participants received partial compensation for their involvement in the study in the form of course credit. The research protocol was approved by the local Ethics Committee.

### Measures

#### SAS-2 (English Original)

The SAS-2 is a 15-item scale designed to assess the level of competitive trait anxiety experienced by athletes before or during competition (Smith et al., 2006). The SAS-2 comprises three factors, namely somatic anxiety, worry, and concentration disruption, each consisting of five items. Participants rate each item on a four-point Likert scale (e.g., “My stomach feels upset”), ranging from 1 (not at all) to 4 (very much). Lower scores indicate lower intensity of competitive anxiety in that aspect, while higher scores suggest a higher likelihood of experiencing that specific type of anxiety. Previous studies have demonstrated that the SAS-2 exhibits a good factor structure and internal consistency, allowing it to be utilized in research regardless of participants’ language (Ramis et al., 2015).

#### Chinese SAS-2

The Chinese version of the SAS-2 was translated by both the authors and bilingual students. The translation process consisted of three parts: translation from English to Chinese, translation from Chinese to English, and independent validation. Initially, the authors translated the English version of the SAS-2 into Chinese. Subsequently, bilingual students proficient in English were invited to translate the Chinese version back into English. Finally, bilingual postgraduate students proficient in English were asked to assess and compare the two English versions to ensure the translated version accurately reflected the original meaning. In cases where discrepancies in meaning arose, the described processes were repeated until all items had consistent and equivalent meaning.

#### Data analysis

In Study 1, we initially compared item scores between the Chinese and English versions of the SAS-2 using a paired-sample *t*-test in a sample of bilingual Chinese university students. Subsequently, we performed a multi-group CFA to assess the measurement invariance of the Chinese and English versions of the SAS-2. Specifically, in this process, we constructed three models as follows: (1) The baseline model, which we initially used to test configural invariance across groups in the original model. (2) The Metric invariance model, built upon the baseline model, introduced the constraint of invariant factor loadings to be equal for both groups. (3) Finally, the Scalar invariance model, an extension of the metric model, imposed the additional constraint of equal item thresholds across groups. One can refer to Millsap (2012) for more detailed analyses of measurement invariance. Multi-group CFA were performed in this study using “lavaan” package (version 0.6–15) in R (version 4.3.0) (Rosseel, 2012) Multiple fit indices were assessed, including the chi-square, RMSEA, TLI, and CFI. Finally, we conducted a paired-samples *t*-test using IBM SPSS Statistics version 22.0.

### Results

#### Paired *t*-tests between Chinese and English SAS-2 item ratings

A paired-sample *t*-test was employed to compare the convergence effect between the Chinese and English versions, as shown in Table 1. As anticipated, both the English and Chinese versions exhibited significant correlations, with all correlations exceeding 0.60. The results of the paired-sample *t*-test indicated no significant difference (all *p* > 0.05) between the Chinese and English versions of the SAS-2 across any of the 15-item scores.

**Table 1:** Paired samples *t*-tests and correlations between Chinese and English SAS-2 item ratings.

Items	*r*	CV (M ± SD)	EV (M ± SD)	*t*
It is hard to concentrate on the game.	0.73^**^	2.04 ± 0.83	2.11 ± 0.95	−0.95
My body feels tense.	0.96^**^	2.74 ± 0.94	2.73 ± 0.91	0.38
I worry that I will not play well.	0.79^**^	2.78 ± 0.95	2.80 ± 0.92	−0.34
It is hard for me to focus on what I am supposed to do.	0.69^**^	2.21 ± 0.91	2.18 ± 0.95	0.43
I worry that I will let others down.	0.92^**^	2.56 ± 0.91	2.54 ± 0.94	0.58
I feel tense in my stomach.	0.84^**^	2.09 ± 1.06	2.15 ± 1.02	−1.06
I lose focus on the game.	0.90^**^	1.99 ± 0.82	1.93 ± 0.83	1.39
I worry that I will not play my best.	0.84^**^	2.55 ± 0.90	2.46 ± 0.92	1.65
I worry that I will play badly.	0.92^**^	2.53 ± 0.90	2.49 ± 0.87	0.90
My muscles feel shaky.	0.74^**^	2.31 ± 101	2.29 ± 0.91	0.30
I worry that I will mess up during the game.	0.72^**^	2.44 ± 0.93	2.38 ± 0.90	0.76
My stomach feels upset.	0.72^**^	2.13 ± 1.01	2.09 ± 0.88	0.59
I cannot think clearly during the game.	0.74^**^	2.07 ± 0.98	2.15 ± 0.91	−1.24
My muscles feel tight because I am nervous.	0.79^**^	2.35 ± 0.95	2.33 ± 0.90	0.35
I have a hard time focusing on what my coach tells me to do.	0.74^**^	1.99 ± 0.84	2.03 ± 0.81	−0.71

EV, English version; CV, Chinese version; **p < 0.001.

#### Measurement invariance analysis

We partitioned the data into two groups, one for the Chinese version of the SAS-2 and the other for the English version of the SAS-2. Subsequently, we conducted a CFA with multiple groups. The results, as presented in Table 2, initially indicate invariance across languages at the Metric invariance level. In other words, the changes in the model fit indices when compared to the baseline model were all less than 0.01 (Cheung and Rensvold, 2002). Furthermore, when we imposed the additional constraint of equal item thresholds, similar results emerged, with changes in CFI, TLI, and RMSEA between Scalar invariance and Metric invariance all remaining below 0.01. The changes in CFI, TLI, and RMSEA were all less than 0.01.

**Table 2:** Measurement invariance of the Chinese SAS-2 across language.

Model	$\chi^{2}$	df	$△\chi^{2}$	_▵_df	CFI	TLI	RMSEA	_▵_CFI	_▵_TLI	_▵_RMSEA
Baseline model	295.08	158			0.948	0.930	0.098			
Metric invariance	310.62	173	15.54	15	0.947	0.936	0.093	0.001	0.006	0.005
Scalar invariance	317.66	185	22.57	27	0.949	0.939	0.089	0.001	0.004	0.004

Baseline model = no invariance; Metric invariance = invariant factor loadings; Scalar invariance = invariant factor loadings and invariant item thresholds.

## Study 2

The aim of Study 2 was to validate the Chinese version of the SAS-2 in a sample of Chinese athlete-students. The study had several objectives. Firstly, we examined the factor structure and reliability of the Chinese version of the SAS-2. Next, we conducted correlation and regression analyses to investigate the convergent and discriminant properties of SAS-2 in relation to other concepts, specifically the State–Trait Anxiety (Spielberger et al., 1971) and Athlete Burnout (Raedeke and Smith, 2001), referencing insights from previous studies (Shou et al., 2017; Wang et al., 2018). Lastly, we explored measurement invariance across genders and score differences in the Chinese version of the SAS-2.

### Method

#### Participants

In Study 2, participants completed the survey on Tencent Questionnaire (https://wj.qq.com/), an online platform like Amazon Mechanical Turk, using their mobile phones. A total of 951 university athlete-students volunteered to participate in Study 2. These participants took this survey on the course for course credit. The protocol for the current study was approved by local Ethics Committee. To ensure data quality, this study sequentially set up two quality check questions in this survey. A total of 92 data were removed based on the results of the two quality testing entries. Moreover, in order to further ensure that participants took their answers seriously, we implemented a criterion based on participants’ response time. As a result, we excluded 24 participants whose response time was less than 200 s, ultimately including 835 athlete-students for analysis.

### Measures

#### SAS-2

The Chinese version of the SAS-2, developed in Study 1, was utilized in the study. The scale comprises three factors, namely somatic anxiety, worry, and concentration disruption, each consisting of five items. Participants were asked to rate 15 items on a four-point Likert scale, with 1 indicating ‘not at all’ and 4 indicating ‘very much’ (e.g., “My stomach feels upset”). Scores for each subscale were determined by averaging the scores of each item within the subscale. The resulting scores ranged from 1 to 4, with lower scores indicating a lower level of competitive anxiety in that specific dimension, while higher scores indicated a higher level of competitive anxiety in that dimension.

#### Athlete Burnout Questionnaire [ABQ]

The ABQ is a multidimensional instrument developed by Raedeke and Smith (2001) to measure athlete perceptions of burnout symptoms. It consists of a total of 15 items that assess three dimensions: Reduced sense of accomplishment, Emotional/physical exhaustion, and Sport devaluation. Participants provided responses to each item using a five-point Likert scale (e.g., “I’m accomplishing many worthwhile things in [sport]”), where 1 corresponded to “almost never,” 2 to “rarely,” 3 to “sometimes,” 4 to “frequently,” and 5 to “almost always.” For each subscale, the dimension score was determined by calculating the mean of the five items within that subscale. Additionally, the total burnout score was calculated by obtaining the mean of all 15 items, incorporating responses from each dimension. The scale has found extensive usage in assessing burnout among youth and college athletes across different countries (Raedeke and Smith, 2001; Sharp et al., 2010). Its Chinese version has also demonstrated satisfactory levels of internal consistency reliability and convergent validity (Liu et al., 2022).

#### State trait anxiety inventory [STAI]

The STAI (Spielberger et al., 1971) was utilized in this study to assess participants’ anxiety symptoms. The scale comprises 40 items that are divided into two subscales: state anxiety (items 1–20) and trait anxiety (items 21–40). Participants rated each item on a scale ranging from 1 to 4(e.g., “I feel calm”), with 1 indicating ‘not at all’ and 4 indicating ‘very much.’ Scores for each item were accumulated after converting the reverse scoring, with higher scores reflecting higher levels of state or trait anxiety. This scale has been validated in a Chinese sample, demonstrating good reliability (Shek, 1993).

#### Data analysis

Study 2 utilized the “lavaan” package (version 0.6-15) in R (version 4.3.0) for conducting CFA, as well as for conducting multigroup confirmatory factor analyses to test measurement invariance (Rosseel, 2012). Additionally, the “lavaanplot” package (Lishinski, 2018) was used to generate the path diagram of the SAS-2. Referring to the original study by Smith et al. (2006), we used the maximum likelihood estimator for the confirmatory factor analyses. Multiple fit indices were assessed, RMSEA, TLI, and CFI. According to Hu and Bentler (1999), an RMSEA value of ≤0.08 indicated an acceptable model fit, while a value of ≤0.05 indicated a good model fit. Additionally, a CFI or TLI value of ≥0.90 indicated adequate model fit. Starting from the perspective of exploring the convergent validity of the SAS-2 with other variables, we conducted a mediation analysis using the PROCESS procedure in SPSS, where the independent variables were STAI, the mediator variable was the SAS-2, and the dependent variable was the ABQ. Descriptive data analysis, reliability analysis, correlation analysis, and regression analysis were performed using IBM SPSS Statistics version 22.0 (IBM Corp., United States).

### Results

#### Descriptive statistics

Descriptive statistics and internal consistency estimate for all measures are presented in Table 3. The Cronbach’s alpha coefficients for all three factors of the SAS-2, as well as the total SAS-2 score, were found to be greater than 0.80. Additionally, all other subscales of the variables of interest demonstrated good reliability coefficients, with the lowest coefficient observed for the Reduced sense of accomplishment subscale in the ABQ (0.62), and the highest coefficient recorded for the ABQ total scale (0.92). Furthermore, we computed the Average Variance Extracted (AVE) and Composite Reliability (CR) for the three SAS-2 factors. The results indicated that for Somatic anxiety, AVE was 0.70, and CR was 0.83. For Worry, AVE was 0.79, and CR was 0.89. Lastly, for Concentration Disruption, AVE was 0.65, and CR was 0.78.

**Table 3:** Descriptive statistics and internal consistency of all measures used in the current study.

Scale	Mean	SD	Alpha	*N* of items
Somatic anxiety	9.07	3.03	0.86	5
Worry	10.55	3.42	0.91	5
Concentration Disruption	8.55	2.91	0.85	5
SAS-2	28.17	8.53	0.94	15
ABQ_RA	14.82	2.93	0.62	5
ABQ_E	13.40	4.13	0.90	5
ABQ_D	13.53	3.95	0.86	5
ABQ	41.76	10.17	0.92	15
STAI-S	45.56	7.28	0.89	20
STAI-T	44.03	8.81	0.81	20

SAS-2, Sport Anxiety Scale-2; ABQ_RA, Athlete Burnout Questionnaire: Reduced sense of accomplishment; ABQ_E, Athlete Burnout Questionnaire: Emotional/physical exhaustion; ABQ_D, Athlete Burnout Questionnaire: Sport devaluation; ABQ, Athlete Burnout Questionnaire; STAI-S, State trait anxiety inventory-state anxiety; STAI-T, State trait anxiety inventory-trait anxiety.

#### Confirmatory factor analysis

**Figure 1:**
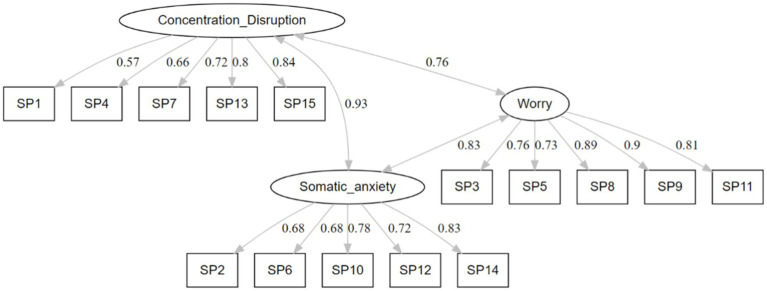
Path diagram of the SAS-2.

The results revealed that the model fit indices for the model were adequate ($\chi^{2}$=403.06, df = 61, *p* < 0.001), and the CFI (0.96) and TLI (0.93) results were both above 0.90, indicating that this model provided a satisfactory fit. The RMSEA (0.08), although not meeting the criteria for a good fit, is still considered acceptable. Figure 1 demonstrates that all items displayed satisfactory factor loadings, which were equal to or greater than 0.40. Additionally, moderate to strong correlations were observed between the three factors, with values ranging from 0.76 to 0.93.

#### Invariance of the SAS-2 across genders

In our study, we assessed the measurement invariance of the Chinese version of the SAS-2 across genders. To achieve this, we first established configural invariance across genders, ensuring that the underlying structure of the scale remained consistent. Subsequently, we conducted tests to examine measurement invariance at both the metric and scalar levels. As shown in Table 4, the changes in CFI, TLI, and RMSEA were all less than 0.01 in both the metric invariance model and the scalar invariance model results (Cheung and Rensvold, 2002). Therefore, these findings provide strong support for the presence of metric and scalar invariance across genders.

**Table 4:** Measurement invariance of the Chinese SAS-2 across gender.

Model	$\chi^{2}$	df	$△\chi^{2}$	_▵_df	CFI	TLI	RMSEA	_▵_CFI	_▵_TLI	_▵_RMSEA
Baseline model	659.23	148			0.943	0.919	0.091			
Metric invariance	659.18	166	35.96^**^	18	0.941	0.926	0.087	0.002	0.007	0.004
Scalar invariance	720.01	178	60.78^***^	30	0.940	0.929	0.085	0.001	0.003	0.002

Baseline mode = no invariance; Metric invariance = invariant factor loadings; Scalar invariance = invariant factor loadings and invariant item thresholds. **p < 0.01; ***p < 0.001.

#### Convergent and discriminant validity

We first analyzed the relationships between the three factors of the SAS-2 and found significant correlations between each pair of factors (Table 5). The correlation coefficients between Somatic Anxiety and Worry were 0.75 (*p* < 0.001), and between Somatic Anxiety and Concentration Disruption were 0.79 (*p* < 0.001). Furthermore, the correlation coefficient between Worry and Concentration Disruption was 0.69 (*p* < 0.001). Subsequently, correlation analyses were conducted to examine the relationships between the factors of the SAS-2 and the criterion scales representing externally related concepts. The results of the correlation analysis are presented in Table 5. The results indicated that all three factors of the SAS-2, namely Somatic anxiety, Worry, and Concentration Disruption, were significantly and positively correlated with all three factors of the ABQ, as well as with State–Trait Anxiety. Notably, all three factors of the SAS-2 had the highest correlations with emotional/physical exhaustion among these external criteria.

**Table 5:** Correlations results between SAS-2 and criterion variables.

Scale	Somatic anxiety	Worry	Concentration disruption
Somatic anxiety	–		
Worry	0.75^**^	–	
Concentration disruption	0.79^**^	0.69^**^	–
ABQ_RA	0.28^**^	0.23^**^	0.32^**^
ABQ_E	0.39^**^	0.31^**^	0.45^**^
ABQ_D	0.36^**^	0.27^**^	0.41^**^
ABQ	0.38^**^	0.28^**^	0.36^**^
STAI-S	0.32^**^	0.28^**^	0.36^**^
STAI-T	0.31^**^	0.28^**^	0.37^**^

ABQ, Athlete Burnout Questionnaire; ABQ_RA, Athlete Burnout Questionnaire: Reduced sense of accomplishment; ABQ_E, Athlete Burnout Questionnaire: Emotional/physical exhaustion; ABQ_D, Athlete Burnout Questionnaire: Sport devaluation; ABQ, Athlete Burnout Questionnaire; STAI-S=State trait anxiety inventory-state anxiety; STAI-T, State trait anxiety inventory-trait anxiety. *p < 0.05. **p < 0.01.

Multiple regression analyses were conducted with the three factors of the SAS-2 as independent variables and the remaining association criteria as dependent variables to assess the unique contribution of each SAS-2 factor in predicting each association criterion (Table 6). Concentration Disruption was found to significant and uniquely predict reduced sense of accomplishment in the ABQ. Both Somatic Anxiety and Concentration Disruption significantly predicted Emotional/Physical Exhaustion and Sport Devaluation, respectively, with Concentration Disruption demonstrating the strongest contribution. Finally, Concentration Disruption was found to uniquely predict state and trait anxiety.

**Table 6:** Summary of regression analyses showing the unique association of each SAS-2 factors (Beta coefficients) with the criterion variables.

Scale	Somatic anxiety	Worry	Concentration disruption
ABQ_RA	0.07	−0.01	0.27^***^
ABQ_E	0.13^*^	−0.05	0.39^***^
ABQ_D	0.14^*^	−0.08	0.35^***^
ABQ	0.13^*^	−0.05	0.37^***^
STAI-S	0.05	0.04	0.30^***^
STAI-T	0.09	0.01	0.29^***^

*p < 0.05. ***p < 0.001.

#### The mediation analysis

**Figure 2:**
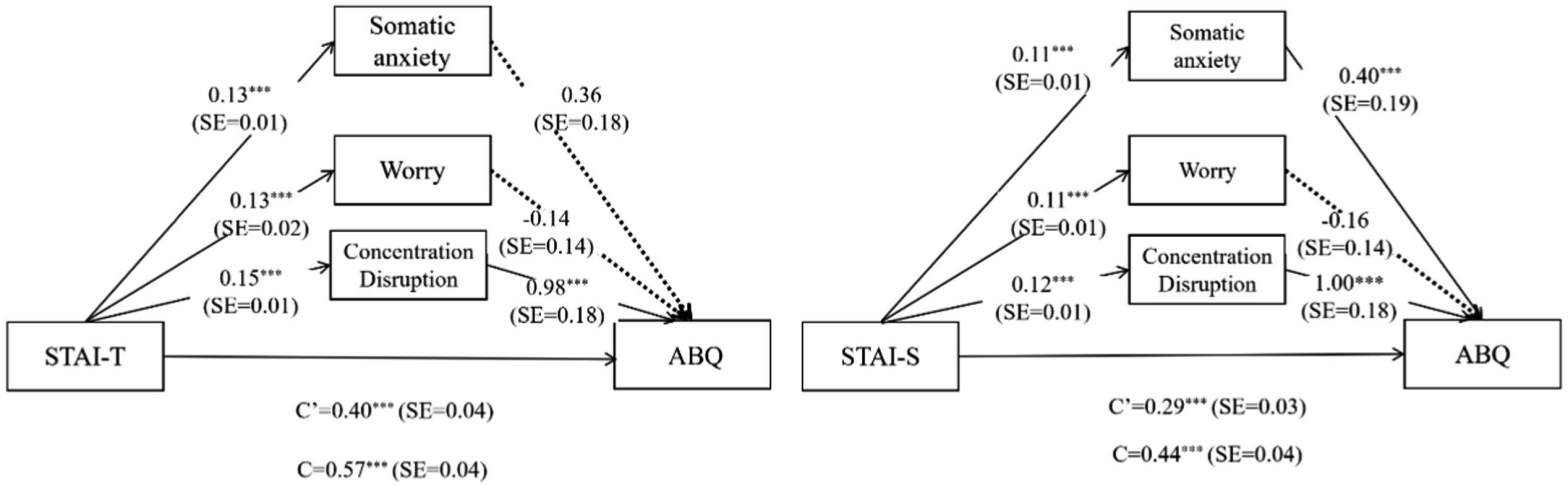
Results of the mediation effects analysis. ***p < 0.001; c’ = direct effect; c = Total effect. The dotted line indicates that the path effect is not significant.

To further scrutinize the convergent validity of the Chinese version of the SAS-2 concerning STAI and ABQ, we performed a series of mediation analyses. In these analyses, we utilized STAI as the independent variable, SAS-2 as the mediator, ABQ as the dependent variable, and included gender as a covariate. The findings indicate that among the three SAS-2 factors, only Concentration Disruption acted as a partial mediator in the relationship between trait anxiety and athlete burnout. Conversely, in the case of the relationship between state anxiety and athletic burnout, both Concentration Disruption and Somatic Anxiety served as partial mediators. This outcome mirrors the regression results presented in Table 6, providing additional support for the association between Concentration Disruption, athlete burnout, and both state and trait anxiety (Figure 2).

#### Differential analysis of gender on the SAS-2

We also compared the differences in the SAS-2 among participants of different genders. Table 7 displays the results of descriptive data and independent samples *t*-tests.

**Table 7:** Independent samples *t*-test results (M ± SD) on the SAS-2 between participants of different genders.

Variables	Male		Female		*t*	*p*
	Mean	SD	Mean	SD		
Somatic anxiety	8.88	3.00	9.35	3.06	−2.21^*^	0.028
Worry	10.24	3.42	11.00	3.38	−3.17^**^	0.002
Concentration disruption	8.52	2.98	8.60	2.82	−0.38	0.707
SAS-2	27.64	8.65	28.95	8.30	−2.18^*^	0.030

*p < 0.05. **p < 0.01.

It was found that female college athletes scored significantly higher on Somatic anxiety (*t* = −2.21, *df* = 833, *p* < 0.05) and Worry (*t* = −3.17, *df* = 833, *p* < 0.01) than male college athletes. The difference in scores on Concentration Disruption between college athletes of different genders was not significant. Lastly, female student-athletes also scored higher than male student-athletes on the SAS-2 total score (*t* = −2.18, *df* = 833, *p* < 0.05).

## Discussion

The current study aimed to translate the SAS-2 into a bilingual sample and conduct cross-validation in a sample of college athletes. In Study 1, we developed the Chinese version of the SAS-2. The bilingual validation results indicate that the meaning of the Chinese version of SAS-2 aligns consistently with the English version of SAS-2, and measurement invariance exists in the English and Chinese versions. In Study 2, we assessed the factor structure of the Chinese version of the SAS-2 and examined its convergent and divergent relationships with other conceptually related criteria. The findings indicated that the Chinese version of the SAS-2 successfully replicated the original three-factor model with good construct validity. Additionally, the regression results highlighted the unique contribution of the SAS-2 factors in predicting other relevant conceptual criteria. Moreover, measurement invariance was observed for the SAS-2 across gender. Lastly, female student-athletes exhibited significantly higher scores than their male counterparts on the SAS-2 Somatic Anxiety and Worry subscales.

More specifically, the Chinese SAS-2 scales also exhibited adequate to good internal consistency. The total scores of the SAS-2 had high reliability (α=0.94), like previous studies conducted with English-speaking samples [α=0.91; Smith et al. (2006)] and Brazilian samples (α=0.88; Silva-Rocha et al. (2019)). Consistent with the original study (Smith et al., 2006), the confirmatory factor analyses also supported the three-factor model of the Chinese version of the SAS-2, with factor loadings ranging between 0.57 and 0.90 for all items. The results of the model fit, on the other hand, indicated that the model fit of the data was generally acceptable (CFI = 0.96, TLI = 0.934, RMSEA = 0.082), as recommended by Bentler and Bonett (1980). When the TLI is greater than 0.90, it indicates an acceptable fit, and when both the CFI and TLI are greater than 0.95, it suggests that the model fit to the data is generally good overall (Hu and Bentler, 1999). Although the current study resulted in RMSEA values around 0.080, previous studies have concluded that RMSEA values between 0.05 and 0.08 are considered acceptable, while values between 0.08 and 0.1 are considered marginal (Fabrigar et al., 1999). Hence, these findings imply that the Chinese version of the SAS-2 not only exhibits strong reliability but also demonstrates sound construct validity, making it a valuable tool for application among Chinese student-athletes.

The present study also examined the convergent and discriminant validity of the SAS-2. Convergent validity was established by demonstrating significant correlations between the SAS-2 and other conceptually relevant scales. Correlation analyses revealed significant correlations between somatic anxiety on the SAS-2 and all three subscales of the ABQ, in addition to state and trait anxiety (ranging from 0.28 to 0.39). Similarly, worry exhibited significant correlations with all three ABQ subscales as well as state and trait anxiety (ranging from 0.23 to 0.31). Importantly, Concentration Disruption demonstrated the highest coefficients among these correlated variables, ranging from 0.32 to 0.45. This finding aligns with prior research (Raedeke and Smith, 2001; Liu et al., 2022). For instance, in Liu et al. (2022) study, it was observed that all three factors of the ABQ exhibited significant positive correlations with both worry and Concentration Disruption. Notably, Concentration Disruption displayed the highest correlation with all three factors of the ABQ. Finally, consistent with previous findings, we found that the three factors of the SAS-2 were significantly correlated with trait and state anxiety (Smith et al., 1995; Liu et al., 2022). Thus, the SAS-2 exhibits a shared variance with the general anxiety measure, indicating a degree of commonality between the two constructs.

The SAS-2 scales also evidenced discriminant validity. Regression analyses found that somatic anxiety significantly predicted emotional/physical exhaustion and sport devaluation of the ABQ, but not Reduced Sense of Accomplishment. Furthermore, somatic anxiety did not significantly predict state and trait anxiety. Worry, on the other hand, failed to significantly predict either the three factors of the ABQ or state and trait anxiety. Finally, we found that concentration disruption significantly predicted all three factors of the ABQ, as well as state and trait anxiety. Concentration disruption alone explained a significant amount of variance in the reduced sense of accomplishment and state and trait anxiety. Additionally, concentration disruption was also the strongest predictor of the emotional/physical exhaustion and sport devaluation variables of the ABQ. In summary, our findings contribute to a deeper understanding of the interplay between the Chinese version of the SAS-2 and these associated measures, offering fresh insights into the convergent and discriminant validity of the SAS-2 in cross-cultural settings.

Additionally, we explored differences in measurement invariance and scores on the three factors of the SAS-2 among college athletes of varying genders. The findings revealed that there was measurement invariance in the Chinese version of the SAS-2 across genders. Furthermore, female college athletes scored significantly higher than their male counterparts on measures of somatic anxiety and worry. This finding is consistent with previous research (Ramis et al., 2015; Correia and Rosado, 2019; Rice et al., 2019). For example, a previous study of a sample of athletes from three different countries also found that females scored significantly higher on worry than males (Ramis et al., 2015). Rice et al. (2019) conducted a systematic regression and meta-analysis of the existing literature and then found that female athletes had higher rates of anxiety and depression than male athletes. Considering the relationship between sport anxiety and well-being and performance, coaches should target female athletes to provide appropriate measures to reduce their sport anxiety levels.

## Limitations and future directions

This study exhibits certain limitations. Firstly, our sample consisted of college students in sports, and we did not examine the psychometric properties of the SAS-2 in a younger adolescent sample. Therefore, future research could focus on examining the reliability and validity of the Chinese version of the SAS-2 in younger samples. Additionally, the current study did not measure participants’ well-being and athletic performance to explore the relationship between the SAS-2 and college athletes’ overall well-being and performance. Consequently, further research could delve into the association between sports anxiety, mental health, and related variables in college athletes using the SAS-2. In addition to the SAS-2, future studies could incorporate objective physiological measures such as galvanic skin response and blood lactate levels to collect comprehensive psychological and physiological data on athletes. For instance, previous research has indicated that elevated blood lactate levels are linked to decreased attention and working memory (Coco et al., 2020). This combined dataset could serve as a valuable resource for enhancing athletic performance, leveraging biofeedback training in conjunction with psychophysiological responses (Pusenjak et al., 2015).

In summary, the present findings indicate that the Chinese version of the SAS-2 exhibits a sound factor structure and reliable internal consistency, as well as measurement invariance across languages and genders. Furthermore, our results provide support for the convergent and discriminant validity of the SAS-2, as evidenced by its associations with various anxiety descriptors and athlete burnout measures in a sample of Chinese student-athletes.

## Data availability statement

The original contributions presented in the study are included in the article/supplementary materials, further inquiries can be directed to the corresponding author.

## Ethics statement

The studies involving humans were approved by the local Ethics Committee (School of General Education, Chongqing Institute of Engineering). The studies were conducted in accordance with the local legislation and institutional requirements. Written informed consent for participation in this study was provided by the participants’ legal guardians/next of kin.

## Author contributions

JZ: Conceptualization, Data curation, Formal analysis, Visualization, Writing – original draft, Writing – review & editing. ZZ: Conceptualization, Data curation, Formal analysis, Supervision, Writing – original draft, Writing – review & editing. SP: Supervision, Writing – review & editing. AV: Supervision, Writing – review & editing. RB: Supervision, Writing – review & editing. WT: Supervision, Writing – review & editing.

## References

[ref1] Bentler P. M. Bonett D. G. (1980). Significance tests and goodness of fit in the analysis of covariance structures. Psychol. Bull. 88, 588–606. doi: 10.1037/0033-2909.88.3.588

[ref2] Brand H. J. Hanekom J. D. Scheepers D. (1988). Internal consistency of the sport competition anxiety test. Percept. Mot. Skills 67, 441–442. doi: 10.2466/pms.1988.67.2.441, PMID: 3217190

[ref3] Cheatham T. Rosentswieg J. (1982). Validation of the sport competition anxiety test. Percept. Mot. Skills 55, 1343–1346. doi: 10.2466/pms.1982.55.3f.1343

[ref4] Cheung G. W. Rensvold R. B. (2002). Evaluating goodness-of-fit indexes for testing measurement invariance. Struct. Equ. Model. 9, 233–255. doi: 10.1207/S15328007SEM0902_5

[ref5] Cho S. Choi H. Eklund R. C. Paek I. (2018). Validation and reliability of the Korean Version of the Sport Anxiety Scale-2. J. Hum. Kinet. 61, 217–225. doi: 10.1515/hukin-2017-0138, PMID: 29599874 PMC5873351

[ref6] Coco M. Buscemi A. Ramaci T. Tusak M. Corrado D. D. Perciavalle V.. (2020). Influences of blood lactate levels on cognitive domains and physical health during a sports stress. Brief review. Int. J. Environ. Res. Public Health 17:9043. doi: 10.3390/ijerph17239043, PMID: 33291577 PMC7729439

[ref7] Correia M. Rosado A. (2019). Anxiety in athletes: gender and type of sport differences. Int. J. Psychol. Res. 12, 9–17. doi: 10.21500/20112084.3552, PMID: 32612783 PMC7110169

[ref8] Di Corrado D. Buscemi A. Magnano P. Maldonato N. M. Tusak M. Coco M. (2021). Mood states and performance in elite canoe polo players: the mediating role of stress. Int. J. Environ. Res. Public Health 18:4494. doi: 10.3390/ijerph18094494, PMID: 33922639 PMC8122864

[ref9] Eysenck M. W. Calvo M. G. (1992). Anxiety and performance: the processing efficiency theory. Cognit. Emot. 6, 409–434. doi: 10.1080/02699939208409696

[ref10] Fabrigar L. R. Wegener D. T. MacCallum R. C. Strahan E. J. (1999). Evaluating the use of exploratory factor analysis in psychological research. Psychol. Methods 4, 272–299. doi: 10.1037/1082-989X.4.3.272

[ref11] Grossbard J. R. Cumming S. P. Standage M. Smith R. E. Smoll F. L. (2007). Social desirability and relations between goal orientations and competitive trait anxiety in young athletes. Psychol. Sport Exerc. 8, 491–505. doi: 10.1016/j.psychsport.2006.07.009

[ref12] Grossbard J. R. Smith R. E. Smoll F. L. Cumming S. P. (2009). Competitive anxiety in young athletes: differentiating somatic anxiety, worry, and concentration disruption. Anxiety Stress Coping 22, 153–166. doi: 10.1080/10615800802020643, PMID: 18937102

[ref13] Hashim H. A. Shaharuddin S. S. Hamidan S. Grove J. R. (2017). A multisample analysis of psychometric properties for the Malaysian adapted Sport Anxiety Scale-2 among youth athletes. Psychol. Rep. 120, 141–157. doi: 10.1177/0033294116685868, PMID: 28558530

[ref14] Hu L.-t. Bentler P. M. (1999). Cutoff criteria for fit indexes in covariance structure analysis: conventional criteria versus new alternatives. Struct. Equ. Model. Multidiscip. J. 6, 1–55. doi: 10.1080/10705519909540118

[ref15] Krohne H. W. Hindel C. (1988). Trait anxiety, state anxiety, and coping behavior as predictors of athletic performance. Anxiety Res. 1, 225–234. doi: 10.1080/08917778808248721

[ref16] Lishinski A. (2018). lavaanPlot: Path diagrams for Lavaan models via DiagrammeR. R package version 0.5.1.

[ref17] Liu H. Wang X. Wu D.-H. Zou Y.-D. Jiang X.-B. Gao Z.-Q.. (2022). Psychometric properties of the Chinese translated athlete burnout questionnaire: evidence from Chinese collegiate athletes and elite athletes. Front. Psychol. 13:823400. doi: 10.3389/fpsyg.2022.823400, PMID: 35602744 PMC9120922

[ref18] Maloney E. A. Sattizahn J. R. Beilock S. L. (2014). Anxiety and cognition. Wiley Interdiscip. Rev. Cogn. Sci. 5, 403–411. doi: 10.1002/wcs.1299, PMID: 26308653

[ref19] Martens R. (1977). Sport competition anxiety test. Human Kinetics Publishers.

[ref20] Martens R. Burton D. Rivkin F. Simon J. (1980). “Reliability and validity of the competitive state anxiety inventory (CSAI)” in *Psychology of motor behavior and sport*, 1979. eds. Nadeau C. H. Newell K. M. Roberts G. C. (Champaign, IL: Human Kinetics), 91–99.

[ref21] Martens R. Burton D. Vealey R. S. Bump L. A. Smith D. E. (1990). Development and validation of the competitive state anxiety inventory-2. Compet. Anxiety Sport 3, 117–190.

[ref22] Millsap R. E. (2012). Statistical approaches to measurement invariance. New York, NY Routledge

[ref23] Pusenjak N. Grad A. Tusak M. Leskovsek M. Schwarzlin R. (2015). Can biofeedback training of psychophysiological responses enhance athletes’ sport performance? A practitioner’s perspective. Phys. Sportsmed. 43, 287–299. doi: 10.1080/00913847.2015.1069169, PMID: 26200172

[ref24] Putra M. F. P. Guntoro T. Wandik Y. Ita S. Sinaga E. Hidayat R.. (2021). Psychometric properties at Indonesian version of the sport anxiety Scale-2: testing on elite athletes of Papua, Indonesian. Int. J. Hum. Mov. Sports Sci. 9, 1477–1485. doi: 10.13189/saj.2021.090645

[ref25] Raedeke T. D. Smith A. L. (2001). Development and preliminary validation of an athlete burnout measure. J. Sport Exerc. Psychol. 23, 281–306. doi: 10.1123/jsep.23.4.281, PMID: 28682196

[ref26] Ramis Y. Torregrosa M. Viladrich C. Cruz J. (2010). Adaptation and validation of the Spanish version of the Sport Anxiety Scale SAS-2 for young athletes. Psicothema 22, 1004–1009. PMID: 21044545

[ref27] Ramis Y. Viladrich C. Sousa C. Jannes C. (2015). Exploring the factorial structure of the Sport Anxiety Scale-2: invariance across language, gender, age and type of sport. Psicothema 27, 174–181. doi: 10.7334/psicothema2014.263, PMID: 25927698

[ref28] Rice S. M. Gwyther K. Santesteban-Echarri O. Baron D. Gorczynski P. Gouttebarge V.. (2019). Determinants of anxiety in elite athletes: a systematic review and meta-analysis. Br. J. Sports Med. 53, 722–730. doi: 10.1136/bjsports-2019-100620, PMID: 31097452 PMC6579501

[ref29] Rosseel Y. (2012). lavaan: an R package for structural equation modeling. J. Stat. Softw. 48, 1–36. doi: 10.18637/jss.v048.i02

[ref30] Sharp L.-A. Woodcock C. Holland M. Duda J. Cumming J. (2010). Validation of the Athlete Burnout Questionnaire with youth athletes. J. Sport Exerc. Psychol. 32, 218–219.

[ref31] Shek D. T. (1993). The Chinese version of the state-trait anxiety inventory: its relationship to different measures of psychological well-being. J. Clin. Psychol. 49, 349–358. doi: 10.1002/1097-4679(199305)49:3<349::AID-JCLP2270490308>3.0.CO;2-J, PMID: 8315037

[ref32] Shou Y. Sellbom M. Han J. (2017). Evaluating the construct validity of the levenson self-report psychopathy scale in China. Assessment 24, 1008–1023. doi: 10.1177/1073191116637421, PMID: 26969688

[ref33] Silva-Rocha V. V. de Sousa D. A. Osório F. L. (2019). Psychometric properties of the Brazilian version of the Sport Anxiety Scale-2. Front. Psychol. 10:806. doi: 10.3389/fpsyg.2019.00806, PMID: 31040807 PMC6477035

[ref34] Smith R. E. Smoll F. L. Barnett N. P. (1995). Reduction of children’s sport performance anxiety through social support and stress-reduction training for coaches. J. Appl. Dev. Psychol. 16, 125–142. doi: 10.1016/0193-3973(95)90020-9

[ref35] Smith R. E. Smoll F. L. Cumming S. P. Grossbard J. R. (2006). Measurement of multidimensional sport performance anxiety in children and adults: the Sport Anxiety Scale-2. J. Sport Exerc. Psychol. 28, 479–501. doi: 10.1123/jsep.28.4.479

[ref36] Smith R. E. Smoll F. L. Schutz R. W. (1990). Measurement and correlates of sport-specific cognitive and somatic trait anxiety: the sport anxiety scale. Anxiety Res. 2, 263–280. doi: 10.1080/08917779008248733

[ref37] Spielberger C. D. (2013). Anxiety and behavior. New York Academic Press.

[ref38] Spielberger C. D. Gonzalez-Reigosa F. Martinez-Urrutia A. Natalicio L. F. Natalicio D. S. (1971). The state-trait anxiety inventory. Interam. J. Psychol. 5, 145–158.

[ref39] Tomczak M. Kleka P. Walczak A. Bojkowski Ł. Gracz J. Walczak M. (2022). Validation of Sport Anxiety Scale-2 (SAS-2) among Polish athletes and the relationship between anxiety and goal orientation in sport. Sci. Rep. 12:12281. doi: 10.1038/s41598-022-16418-6, PMID: 35853925 PMC9296646

[ref40] Wang M.-C. Shou Y. Deng Q. Sellbom M. Salekin R. T. Gao Y. (2018). Factor structure and construct validity of the Levenson Self-Report Psychopathy Scale (LSRP) in a sample of Chinese male inmates. Psychol. Assess. 30, 882–892. doi: 10.1037/pas0000537, PMID: 29565613

[ref41] Weidman A. C. Fernandez K. C. Levinson C. A. Augustine A. A. Larsen R. J. Rodebaugh T. L. (2012). Compensatory internet use among individuals higher in social anxiety and its implications for well-being. Personal. Individ. Differ. 53, 191–195. doi: 10.1016/j.paid.2012.03.003, PMID: 22791928 PMC3392122

[ref42] Woodman T. Hardy L. (2003). The relative impact of cognitive anxiety and self-confidence upon sport performance: a meta-analysis. J. Sports Sci. 21, 443–457. doi: 10.1080/0264041031000101809, PMID: 12846532

